# The impact of antibiotic therapy in psoriasis patients with active streptococcal infection: A prospective study

**DOI:** 10.1111/1346-8138.17645

**Published:** 2025-02-03

**Authors:** D. Bonciani, C. Della Bella, A. Corrà, A. Galano, G. Vaggelli, S. Tapinassi, A. Grassi, E. Mantengoli, A. Coi, L. Quintarelli, E. B. Mariotti, C. Aimo, V. Ruffo di Calabria, E. Garufi, V. Volpi, A. Verdelli, G. M. Rossolini, A. Bartoloni, M. M. D'Elios, M. Caproni

**Affiliations:** ^1^ Section of Dermatology, Department of Health Sciences University of Florence Florence Italy; ^2^ Department of Experimental and Clinical Medicine University of Florence Florence Italy; ^3^ Microbiology and Virology Unit Careggi University Hospital Florence Italy; ^4^ Infectious and Tropical Diseases Unit Azienda Ospedaliero‐Universitaria Careggi Florence Italy; ^5^ Institute of Clinical Physiology National Research Council Pisa Italy; ^6^ Rare Skin Diseases Unit, P.O. Piero Palagi, Azienda USL Toscana Centro University of Florence Florence Italy

**Keywords:** antibiotic therapy, psoriasis, Psoriasis Area and Severity Index, streptococcal infection, streptococcus pharyngeal infection

## Abstract

The association between psoriasis and streptococcal infection has been widely explored in both children and adults. However, the exact impact of Streptococcus pharyngeal infection on the course of psoriasis is not fully comprehended. This study explored the impact of Streptococcus pharyngeal infection on psoriasis and investigated the effectiveness of systemic antibiotic therapy in conjunction with standard topical treatment for psoriatic patients with concomitant streptococcal throat infection. The research involved 115 patients with mild‐to‐moderate psoriasis, clinically assessed using the Psoriasis Area and Severity Index (PASI). Patients with active streptococcal infection were administered adjunctive systemic antibiotic therapy along with standard local treatment for psoriasis, while psoriasis patients without evidence of infection received the local topical treatment only. Streptococcal infections were more common in psoriasis patients compared to healthy controls. A group of psoriasis patients with active streptococcal throat infections, treated with antibiotics in addition to standard topical psoriasis therapy, did not show any difference in PASI score reduction compared to those without evidence of active infection. While our study did not show a statistically significant reduction in PASI scores in psoriasis patients with streptococcal throat infections treated with antibiotics, it highlights the complex interaction between infection and psoriasis. Larger studies with longer follow‐up may better clarify this relationship, contributing to stronger evidence for or against the use of antibiotics in managing psoriasis triggered by streptococcal infections.

## 1 INTRODUCTION

Psoriasis is a chronic‐relapsing, inflammatory, systemic disease with an estimated prevalence of around 2%–3% of the population. The skin is the primarily affected organ, mostly with erythemato‐squamous plaques defining the most common phenotype, named psoriasis vulgaris. Guttate psoriasis is an eruptive form with smaller lesions, appearing preferentially in pediatric patients. Although the pathogenesis is still not fully understood, evidence supports the role of a variety of bacterial, viral, and fungal infections in psoriasis induction or exacerbation.^1^, ^2^ For example, bacterial ribosomal DNA was detected in blood samples of guttate and plaque psoriasis patients.^3^ Several lines of research show that among the bacterial triggers, group A β‐hemolytic streptococcus (*Streptococcus pyogenes*) is the most traditionally associated species to psoriasis. In fact, psoriasis and recurrent or chronic streptococcal tonsillitis share the same risk allele, being both associated with human leukocyte antigen‐C*06:02.^4^, ^5^, ^6^ In addition, β‐hemolytic streptococcus is more likely cultured from psoriasis patients throat swabs than controls,^6^ while in some retrospective and prospective studies the worsening of psoriasis was associated with sore throat or tonsillitis in percentages varying from 33% to 42% of patients. Moreover, 72% of patients with confirmed streptococcal infections reported worsening of the skin disease in a cohort study.^7^, ^8^ The evidence of an association between psoriasis and acute or recurrent streptococcal pharyngitis has been recently extended to the pediatric population.^2^, ^9^ The natural and logical question raised by this amount of evidence is about the effectiveness of antistreptococcal interventions in improving time‐to‐resolution or long‐term control of psoriasis in patients with proven infection. To date, several studies tried to address this concern, but failed to provide solid, combined evidence.^10^ Our aim was to contribute to this field of research by assessing the prevalence of bacterial throat infection among a large cohort of psoriasis patients and the influence of antibiotic therapy in a selected subgroup.

## 2 MATERIALS AND METHODS

This was a prospective, single center, 3‐year observational study conducted in accordance with the Declaration of Helsinki and approved by the local medical ethics committee (Florence, Italy).

### 2.1 Patients' enrolment

Patients with a diagnosis of psoriasis referred to the psoriasis center of the Dermatology clinic of the University of Florence were consecutively enrolled.

The following characteristics were chosen as inclusion criteria: (1) age <80 years old; (2) onset or flare of mild‐to‐moderate psoriasis diagnosed through clinical examination and histopathology; and (3) ability to provide written informed consent.

Exclusion criteria were: (1) pustular or erythrodermic phenotypes of psoriasis; (2) concomitant psoriatic arthritis; (3) history of spontaneous remission of psoriasis, (4) history of antibiotic allergy (5) pregnancy or breastfeeding; and (6) history of other immune‐mediated diseases.

### 2.2 Disease activity

At the time of inclusion in the study, clinical data were collected for each patient, including Psoriasis Area and Severity Index (PASI) scores. A PASI score was also collected at each timepoint.

### 2.3 Evaluation of streptococcal infection

The following procedures were carried out for each patient in order to assess the possible infection: (1) a throat swab, to detect the presence of β‐hemolytic streptococci, and (2) a blood sample for the determination of anti‐streptolysin O (ASO) titer.

As a control, pharyngeal swabs were collected from age‐ and sex‐matched dermatologic, non‐psoriatic patients referred to our site in the same period.

Microbiological analyses were performed to verify the presence of β‐hemolytic streptococcal infection. In the positive cases, the isolated bacteria were compared with each other and with other previously isolated bacteria using high‐resolution genotyping techniques.

Pharyngeal swabs were cultured both on Columbia blood agar plates (incubation at 37°C in air enriched with 5% CO_2_) and on Group A selective agar plates (incubation at 37°C in anaerobiosis). Suspected β‐hemolytic colonies isolated on plates were identified by the matrix‐assisted laser desorption ionization‐time of flight (MALDI‐TOF) method. In addition, the latex agglutination test (LAT) was performed for Lancefield group definition.^11^

Antimicrobial susceptibility to penicillin G, erythromycin, and clindamycin was evaluated using the Kirby‐Bauer method^12^ in accordance with European Committee on Antimicrobial Susceptibility Testing (EUCAST) protocols (www.eucast.org). The reference strain *Streptococcus pneumoniae* ATCC 49619 was included for quality control. Results were interpreted in accordance with the EUCAST clinical breakpoints (version 9.0, 2019).

### 2.4 Patients' treatment and follow‐up

Patients with β‐hemolytic streptococcal throat swab positivity were considered positive for streptococcal infection.

Subsequently, the patients with streptococcal infection received both topical psoriasis treatment and oral antibiotic therapy while patients without streptococcal infection (including those with positive ASO titer and negative throat swab) were only treated with conventional psoriasis therapy. Topical therapy consisted of a combination of betamethasone and calcipotriol in gel.

The antistreptococcal antibiotic therapy, recommended by the infectious diseases specialist, was set up according to the results of susceptibility testing, a history of allergies or other adverse events as well as previous antibiotic treatments. Treatment schedules included amoxicillin (1 g tid for 6 days), clarithromycin (500 mg bid for 7 days), and clindamycin (300 mg tid for 10 days).

All patients were evaluated during enrollment visit (T0) and after 1 month (T1). Then, follow‐up visits were carried out at 6, 12, and 18 months from the beginning of the therapy (respectively T2, T3, and T4), to evaluate clinical response and the number and severity of recurrences. The measured outcomes were mean PASI score in both patient groups at T1, T2, T3 and T4 as well as the percentage of patients reaching a PASI sore of ≥75% (PASI75) or ≥90% and (PASI90) at every timepoint.

### 2.5 Statistical analysis

At baseline, the differences in demographics (age and gender) in throat swabs (defined as positive or negative) or antibodies (defined as positive titer >200 IU/mL and negative titer <200 IU/mL) were evaluated in four subtypes of psoriasis (vulgaris, guttate, palmoplantar, and inverse) using Fisher's exact test for categorical variables and the Student's *t*‐test for continuous variables. For continuous variables, mean with 95% confidence intervals (95% CI) are reported in the text. A multivariate analysis by logistic regression was performed when age and/or gender resulted as significantly associated with either throat swabs or titer and the investigated psoriasis subtypes.

Changes in PASI scores at the 1‐, 6‐, 12‐, and 18‐month follow‐ups were compared among groups of subjects based on their ASO titer status (positive or negative) and their throat swab results (positive or negative). Differences in PASI scores between subjects undergoing streptococcal therapy and subjects not receiving therapy were assessed with the Student's *t*‐test.

In all analyses, a two‐sided *p* value of <0.05 was considered statistically significant. Statistical analyses were performed using STATA software StataCorp, College Station, TX, USA.

## 3 RESULTS

A total of 155 patients with psoriasis were enrolled. Among them, 86 (55.5%) were males and 69 (45.5%) were females. The mean ± standard deviation (SD) age at the time of enrollment was 42.9 years ± 16.7 years. Concerning the clinical presentation, 95 patients (61.3%) were diagnosed with psoriasis vulgaris (chronic plaque psoriasis), whereas 36 patients (23.2%) presented with guttate psoriasis; five patients (3.2%) presented lesions compatible with the palmo‐plantar variant, while two patients (1.3%) showed the inverse variant. Finally, some patients with a previous diagnosis of psoriasis vulgaris experienced an acute guttate flare and were included in a specific group (*N* = 17, 11.0%). No significant difference in gender distribution among patients with different types of psoriasis was detected.

Regarding the streptococcal infection, 39 psoriasis patients (25.5%) had a positive throat swab while 80 (51.6%) presented positive ASO titer (>200 IU/mL).

Patients with psoriasis vulgaris were significantly older than patients with other psoriasis subtypes (*p* < 0.0001). On the other hand, patients with guttate psoriasis showed a mean age significantly lower than the other groups (*p* = 0.0001) (Table 1). No statistical results emerged concerning the age of patients with palmo‐plantar or inverse psoriasis, or for those with psoriasis vulgaris and an acute guttate flare.

**TABLE 1:** Distribution of age, sex, serological al microbiological data grouped according to psoriasis phenotypes.

	Sample size (*n* = 155)	Psoriasis vulgaris			Guttate psoriasis			Palmo‐plantar psoriasis			Inverse psoriasis			Psoriasis vulgaris + guttate flare		
		Y (*n* = 95)	N (*n* = 60)	*p*	Y (*n* = 36)	N (*n* = 119)	*p*	Y (*n* = 5)	N (*n* = 150)	*p*	Y (*n* = 2)	N (*n* = 153)	*p*	Y (n = 17)	N (*n* = 138)	*p*
Age Mean ± SD (years)	42.9 ± 16.7	48.1 ± 16.6	35.0 ± 13.4	<0.001	34.0 ± 13.2	46.1 ± 16.7	0.0001	41.6 ± 15.3	42.9 ± 16.8	ns	37.5 ± 0.7	42.9 ± 16.8	ns	38.5 ± 16.8	43.4 ± 16.6	ns
Males (*n*, %)	86 55.5	58 61.0	28 46.7	ns	15 41.6	69 57.9	ns	2 40.0	84 56.0	ns	1 50.0	85 55.6	ns	10 58.8	76 55.1	ns
Females (*n*, %)	69 45.5	37 39.0	32 53.3		21 58.3	50 42.0		3 60.0	66 44.0		1 50.0	68 44.4		7 41.2	62 44.9	
Negative throat swab (*n*, %)	114 74.5	78 83.0	36 61.0	0.002	21 59.0	91 79.8	ns	3 60.0	112 74.7	ns	2 100.0	112 74.2	ns	11 64.7	103 75.7	ns
Positive throat swab (*n*, %)	39 25.5	16 17.0	23 39.0		15 41.0	23 20.2		2 40.0	38 25.3		0 0.0	39 25.8		6 35.3	33 24.3	
ASO titer <200 IU/mL	75 48.4	51 53.7	24 40.0	0.097	13 32.5	62 53.9	ns	1 20.0	74 49.3	ns	2 100.0	73 47.7	ns	10 58.8	65 47.1	ns
ASO titer >200 IU/mL	80 51.6	44 46.3	36 60.0		23 67.5	53 46.1		4 80.0	76 50.7		0 0.0	80 52.3		7 41.2	73 52.9	

Abbreviations: ASO, anti‐streptolysin O; N, no; ns, not statistically significant; SD, standard deviation; Y, yes.

Some of the patients were lost at follow‐up during the study: 13 cases were lost between T0 and T3, while at the T4 timepoint a total of 26 patients were lost.

A total of 252 subjects were enrolled in the healthy control (HC) group. Among them, 102 (40.0%) were males and 150 (60.0%) were females. The mean age of the HC group was 44.5 ± 15.4 years. Streptococcal throat swab resulted positive in 32 of the 252 patients. The proportion was significantly lower than in the group of psoriatic patients (12.7% vs. 20.4%, *p* = 0.039).

### 3.1 Throat swab and ASO titer (Table 2)

**TABLE 2:** PASI score measured at each timepoint in patients grouped on the basis of positivity of the ASO titer.

PASI	ASO titer						Antistreptococcal therapy (for patients with ASO titer > 200 IU/mL)					
	>200 IU/mL			<200 IU/mL			Y (positive throat swab)			N (negative throat swab)		
	*n*	PASI score: Mean ± SD	*p*	*n*	PASI score: Mean ± SD	*p*	*n*	PASI score: Mean ± SD	*p*	*n*	PASI score: Mean ± SD	*p*
T0 (mean ± SD)	80	5.92 ± 2.95		75	6.47 ± 3.15		22	5.94 ± 3.50		58	5.92 ± 2.75	
T1 (1 month) (mean ± SD)	80	3.26 ± 3.09	<0.0001	75	3.30 ± 3.00	<0.0001	22	2.96 ± 2.38	0.002	58	3.38 ± 3.33	<0.0001
T2 (6 months) (mean ± SD)	80	1.82 ± 1.87	0.0005	75	1.89 ± 1.85	0.0007	22	1.59 ± 1.73	0.035	58	1.91 ± 1.93	0.004
T3 (12 months) (mean ± SD)	75	1.72 ± 2.21	0.761	67	1.78 ± 2.42	0.760	21	1.86 ± 3.41	0.743	54	1.67 ± 1.56	0.473
T4 (18 months) (mean ± SD)	69	1.20 ± 1.45	0.100	60	1.62 ± 2.33	0.706	19	1.00 ± 1.37	0.311	50	1.28 ± 1.48	0.195
Mean PASI reduction from T0 to T4 (%)		78.3%			79.1%			84.4%			76.0%	

*Note:* Patients with an ASO >200 IU/mL and positive throat swab were then assigned to the group receiving topical psoriasis treatment associated with systemic antibiotic therapy, while patients with positive ASO titer and negative throat swab received antipsoriatic local therapy alone. Statistical analysis (*p*) was performed to assess the significance of mean PASI difference from a follow‐up to next one.

Abbreviations: ASO, anti‐streptolisin O; N, no; PASI, Psoriasis Area and Severity Index; SD, standard deviation; Y, yes.

No statistical gender difference was found in analysis of the results of throat swabs and ASO titers.

In the psoriasis vulgaris patient group, throat swabs were positive in 16 cases and negative in 114 cases. In the same group, the ASO titer resulted in >200 IU/mL in 44 cases and <200 IU/mL in 51 cases.

Among patients with guttate psoriasis, the throat swab tested positive in 15 cases and negative in 21 cases; the ASO titer was positive in 23 cases and negative in 13 subjects.

The five patients of the palmoplantar psoriasis group showed a positive throat swab in two cases, while the other three subjects were negative; ASO titer was >200 IU/mL in four subjects and negative in one subject. Two patients with inverse psoriasis type showed a negative swab and a negative ASO titer.

Six patients (35.3%) had a positive throat swab among patients affected by psoriasis vulgaris with a guttate flare. In the same group, seven patients (41.2%) had an ASO titer of >200 IU/mL.

Subjects with a positive throat swab were significantly younger than those with a negative throat swab (34.90 ± 16.41 vs. 45.94 ± 15.96, *p* = 0.0003). For this reason, age must be considered a confounding factor in the association between the result of throat swab and vulgaris or guttate psoriasis subtypes. In fact, statistical descriptive analysis evidenced an association between a positive throat swab and psoriasis vulgaris which lost its significance when age adjusted. Similarly, subjects with an ASO titer of >200 IU/mL were of a significantly lower age compared to subjects with a titer <200 IU/mL (37.5 ± 14.9 vs. 48.8 ± 16.6, *p* < 0.0001). Age ,therefore, considered a confounding factor of the association between the result of ASO titration and vulgaris and guttate psoriasis subtypes. Analogously, no significant associations between ASO titer and psoriasis subtypes were found after age‐adjustment.

### 3.2 PASI score and ASO titer association analysis

In both ASO‐positive and ASO‐negative subject groups, average PASI score significantly decreased from T0 to T1 and from T1 to T2 (Table 2). The decrease in PASI scores at T3 and T4 did not reach statistical significance in either group. The reduction of PASI score from T0 to T4 was similar (78.3% ± 27.1 vs. 79.1% ± 24.1).

Significant reduction of mean PASI scores from T0 to T1 and from T1 to T2 was evidenced in both ASO‐positive cases treated with antibiotic therapy and ASO‐positive subjects not receiving antibiotics (Table 2).

Finally, PASI percentage reduction from T0 to T4 was more marked for patients treated with antibiotics compared to those not treated (84.4% ± 21.8 vs 76.0% ± 28.7). However, the difference did not reach statistical significance.

The cumulative rate of patients reaching PASI75 or PASI90 at every timepoint did not show any statistical difference between the two groups (Tables 3 and 4).

**TABLE 3:** Proportions of patients reaching PASI75 at each timepoint.

PASI75	Antibiotic therapy		*p*
	Y (*n*, %)	N (*n*, %)	
T1	3 (9.7)	19 (21.6)	ns
T2	18 (58.1)	40 (45.4)	ns
T3	16 (53.3)	47 (56.6)	ns
T4	15 (53.6)	45 (57.7)	ns

Abbreviations: N no; PASI75, Psoriasis Area and Severity Index ≥75% improvement; Y, yes.

**TABLE 4:** Proportions of patients reaching PASI90 at every timepoint.

PASI90	Antibiotic therapy		*p*
	Y *n*, (%)	N *n*, (%)	
T1	1 (3.2)	9 (10.2)	ns
T2	7 (22.6)	29 (33.0)	ns
T3	11 (36.7)	34 (41.0)	ns
T4	10 (35.7)	33 (42.3)	ns

Abbreviation: N, no; ns, not significant; PASI90, Psoriasis Area Severity Index score of ≥90% improvement; Y, yes.

## 4 DISCUSSION

Our study highlighted a significantly higher prevalence of confirmed streptococcal throat colonization in psoriasis patients (25.5%) compared to the control group (12.7%). Similarly, Gudjonsson et al. isolated β‐hemolytic streptococci from about 10% of psoriasis patients complaining of sore throat, while the same bacteria were found in <1% of the controls.^13^ However, our study did not reveal any difference in prevalence of throat colonization between psoriatic males and females.

The diagnostic method used to assess the presence of active streptococcal infection was the MALDI‐TOF technique performed on cultured throat swabs, because the ASO titer cannot discriminate between active and past infection, and is, therefore, no longer considered a relevant diagnostic test alone.^14^, ^15^

To date, no antistreptococcal interventions are recommended in therapeutic guidelines for psoriasis, due to the lack of firm evidence of efficacy.^13^ Nevertheless, antibiotic therapy or tonsillectomy have already been adopted in the clinical practice to reduce psoriasis flares. Several authors in the literature reported efficacy of those interventions, although often through uncontrolled trials with wide heterogeneity in antimicrobial treatments and variable assessment methods of streptococcal infection.^16^, ^17^, ^18^ Therefore, the prescription of antibiotics or tonsillectomy for psoriasis remains controversial. In our study, analysis of the impact of tonsillectomy in a dedicated patient groups was not regarded as ethically possible as it would have represented an invasive treatment with the possibility of complications. However, our results do not support the effectiveness of adjunctive, anti‐streptococcal treatment after 18 months of follow‐up. In fact, patients with streptococcal throat colonization treated with a course of antibiotic therapy experienced a greater mean PASI reduction compared to patients without streptococcal pharyngitis, but the difference was not significant.

Persistent streptococcus superantigen activity has been hypothesized to trigger psoriasis onset or flares. A pathogenetic link between streptococcal immune response and psoriasis was suggested by the finding of circulating T cells reacting to shared sequences of streptococcal M protein and keratin‐derived peptides in psoriatic patients.^19^, ^20^ Consequently, antigen‐presenting cells may expose streptococcal antigens inducing a skin‐homing phenotype in CD8+ cells which then infiltrate skin inducing psoriatic changes following presentation of homologous epitopes by keratinocytes. Moreover, circulating psoriatic cutaneous lymphocyte–associated antigen (CLA) + memory T cells cultured with epidermal cells showed activation following the addition of streptococcal extracts, resulting in increased production of Th1, Th17, and Th22 cytokines.^21^, ^22^

Concerning the limitations of our study, a comparison between oral antibiotic versus placebo in two different arms of patients with positive streptococcal swab would have been an interesting adjunctive investigation, but the low samples size would have compromised their representativeness. Moreover, the local ethics committee did not approve the possibility of a control group obtained by not prescribing antibiotic therapy to patients with proven throat bacterial infection. While the findings do not support a role for antibiotics in reducing psoriasis severity, it should be noted that patients with throat streptococcal infection were not re‐tested to verify the effective eradication of the bacterial reservoir. This does not completely rule out the possibility of benefit for certain subgroups. For instance, a longer treatment duration or different antibiotic regimens might have yielded different results. Pending the availability of solid evidence, clinicians might still consider antibiotics for psoriasis patients with clear streptococcal infection in specific circumstances, especially if infections are recurrent or where there is an unequivocal temporal link between psoriasis flares and episodes of infection.

The correct selection of candidates, however, should include a highly reliable diagnostic assessment to ensure the administration of antistreptococcal therapy only to subjects with proven correlation between infection and psoriasis. In fact, considering the high prevalence of psoriasis, an exaggeratedly wide prescription of antibiotics with negative implications in terms of antibiotic resistance must be avoided. Rapid lesional or circulating lymphocytes activation assays in response to streptococcal antigens may represent an interesting approach for screening as they showed encouraging results.^23^

While our study did not demonstrate a significant difference in PASI scores reduction in psoriasis patients with streptococcal throat infections treated with antibiotics, it highlights the complexity of the interaction between infection and psoriasis. Future studies with larger sample sizes and longer follow‐up may better clarify this relationship, thus contributing to stronger evidence to the limited use of antistreptococcal interventions or, conversely, to question the results of this study.

## FUNDING INFORMATION

The work was funded with grants derived from the “Ricerca Finalizzata” public call, promoted by the Italian Ministry of Health, which stipulated a specific convention with Tuscany Region for the realization of the project. Prof. Marzia Caproni, Prof. Mario Milco D'Elios, Prof. Alessandro Bartoloni and Prof. Gian Maria Rossolini obtained the grant with the project entitled “Targeting streptococcal infections in the management of patients with psoriasis” (project code 16RFAB).

## CONFLICT OF INTEREST STATEMENT

None declared.
